# Efficacy of Topical Alpha Ointment (Containing Natural Henna) Compared to Topical Hydrocortisone (1%) in the Healing of Radiation-Induced Dermatitis in Patients with Breast Cancer: A Randomized Controlled Clinical Trial

**Published:** 2013-12

**Authors:** Mansour Ansari, Farzin Dehsara, Ahmad Mosalaei, Shapour Omidvari, Niloofar Ahmadloo, Mohammad Mohammadianpanah

**Affiliations:** 1 Department of Radiation Oncology, Nemazee Hospital, Shiraz University of Medical Sciences, Shiraz, Iran;; 2 Student Research Committee, Department of Radiation Oncology, Nemazee Hospital, Shiraz University of Medical Sciences, Shiraz, Iran;; 3 Shiraz Institute for Cancer Research, Department of Radiation Oncology, Nemazee Hospital, Shiraz University of Medical Sciences, Shiraz, Iran;; 4 Colorectal Research Center, Faghihi Hospital, Shiraz University of Medical Sciences, Shiraz, Iran

**Keywords:** Radiation-induced dermatitis, Hydrocortisone, Breast cancer, Radiotherapy

## Abstract

**Background: **This two-arm, randomized clinical study aimed to compare efficacy between topical Alpha ointment and topical hydrocortisone cream (1%) in the healing of radiation-induced dermatitis in breast cancer patients.

**Methods: **The inclusion criteria comprised newly pathologically proven, locally advanced breast cancer (treated with modified radical mastectomy followed by sequential adjuvant treatments, including chest wall radiotherapy [45-50.4 Gy]) and grade 2 and/or 3 chest wall dermatitis. The exclusion criteria were comprised of any underlying disease or medications interfering with the wound healing process, previous history of chest wall radiotherapy, and concurrent use of chemotherapy. Sixty eligible patients were randomly assigned to use either topical Alpha ointment (study arm, n=30) or topical hydrocortisone cream (1%) (control arm, n=30) immediately after receiving a total dose of 45-50 Gy chest wall radiotherapy.

**Results:** The mean radiation dose was 49.1 Gy in the control arm and 48.8 Gy in the study arm. The mean dermatitis area was 13.54 cm^2^ in the control arm and 17.02 cm^2^ in the study arm. Topical Alpha ointment was more effective on the healing of radiation-induced dermatitis than was topical hydrocortisone cream (1%) (P=0.001). This effect was significant in the second week (P=0.007). In addition, Alpha ointment decreased the patients’ complaints such as pain (P<0.001), pruritus (P=0.009), and discharge (P=0.010) effectively and meaningfully.

**Conclusion: **Topical Alpha ointment was more effective on the healing of radiation-induced dermatitis than was topical hydrocortisone cream (1%) in our patients with breast cancer.

**Trial Registration Numbers: **IRCT201206099979N1, ACTRN12612000837820

## Introduction

Radiotherapy plays a critical role in the management of breast cancer patients. Over 90% of patients who undergo modified radical mastectomy for their locally advanced disease requiring adjuvant chest wall radiotherapy develop radiation dermatitis. Breast cancer patients receiving chest wall radiotherapy develop acute skin toxicity (radiation dermatitis) during the course of radiotherapy or a short period after the completion of radiotherapy.^1^^,^^2^ Chest wall radiation dermatitis can decrease tolerance for continuing radiotherapy, negatively influence quality of life, postpone treatment, and cause treatment failure.^1^ For the all the research hitherto conducted on the management of radiation-induced dermatitis, a consensus has yet to emerge as to what constitutes the optimal care.^2^

Topical corticosteroids comprise one group of the suggested agents for the treatment of radiation-induced dermatitis. Corticosteroids have anti-inflammatory effects, which may play a crucial role in alleviating patients’ complaints. Recent evidence shows the efficacy of topical corticosteroids in this category.^2^^,^^3^ In addition, other local treatments such as Dexpanthenol, Calendula, and honey ointment have been recommended for treating dermatitis.^4^^,^^5^ Another drug which has newly been introduced for the management of burning and infectious wounds is natural Henna (Lawsonia inermis linn),^6^ with some investigators providing evidence for its antimicrobial and antioxidant properties in wound healing as well.^7^^-^^10^ The data regarding the efficacy of Henna compounds in the management of burn and infected wounds are, however, insufficient, and there are no optimal recommendations for skin care in breast cancer patients suffering radiation dermatitis. This study aimed to compare topical Alpha ointment and topical hydrocortisone cream (1%) in terms of their efficacy in the healing of radiation-induced dermatitis in breast cancer patients undergoing post-mastectomy chest wall radiotherapy.

## Patients and Methods

This study is an open, randomized, controlled, phase II clinical trial. Eligible patients had newly pathologically proven, locally advanced breast cancer (treated with modified radical mastectomy, followed by sequential adjuvant chemotherapy and chest wall radiotherapy [45-50.4 Gy]) and grade 2 and/or 3 radiation-induced dermatitis. Exclusion criteria consisted of any history of collagen vascular diseases, diabetes mellitus, taking any drugs interfering with the wound healing process like systemic steroids, previous history of chest wall radiotherapy, and concurrent use of chemotherapy. All the patients had to sign the consent form before participating in the study. This clinical trial was approved by the local Research Ethics Committee of Shiraz University of Medical Sciences. 

**Figure 1:**
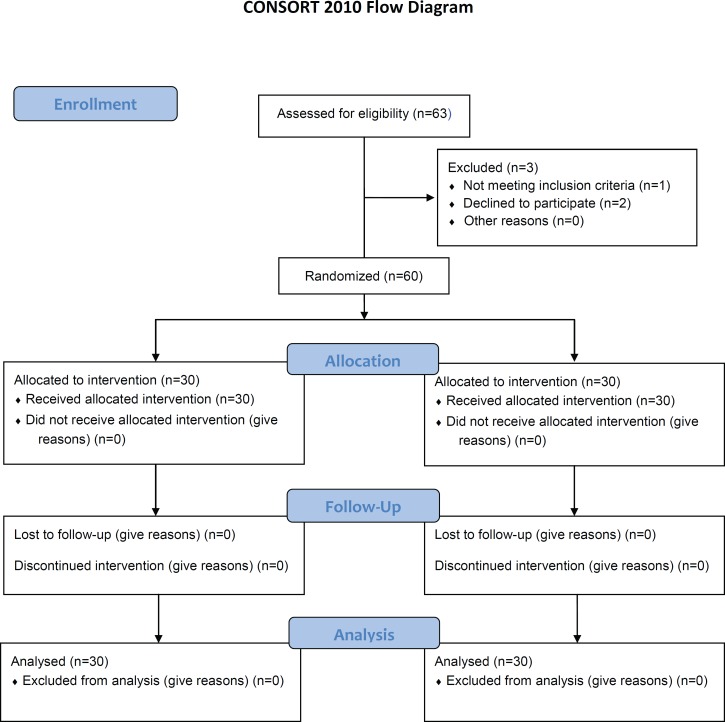
This is a depiction of the CONSORT flow diagram of 60 breast cancer patients, who developed chest wall radiation dermatitis

Between July 2011 and December 2011, sixty-three eligible patients were randomly assigned to received either topical Alpha ointment (study arm, n=32) or topical hydrocortisone cream (1%) (control arm, n=31) immediately after completing post-mastectomy chest wall radiotherapy with a median dose of 45-50 Gy. In addition, all the patients in both arms were recommended to perform daily chest wall simple washing with mild soap. Three patients were excluded from the study: one developed grade IV dermatitis in the first week of intervention and did not meet the inclusion criteria, and two patients developed severe pain following topical application and declined to participate (figure 1).

Dermatitis grade was determined according to the Common Terminology Criteria for Adverse Events (version 4.0). Dermatitis grade 2 was defined as moderate to brisk erythema, patchy moist desquamation mostly confined to skin folds and creases, and moderate edema of the irradiated chest wall. Dermatitis grade 3 was defined as moist desquamation in areas other than skin folds and creases, bleeding induced by minor trauma, or abrasion of the chest wall.^11^ Radiation portals were composed of chest wall fields in all the patients and supraclavicular, post-axillary, and internal mammary fields in most of them. Optimization of the skin dose to reach the prescribed total radiation dose was achieved by treating the chest wall fields in all the patients in both arms with superficial X-ray (120 KV, 10 mA, 2 mm Al filtration, 4 mm Al half-value layer) and with a daily fraction of 1.8-2 Gy (five fractions per week). All the patients had to receive a total dose of 45-50.4 Gy. In the control arm, topical hydrocortisone cream (1%) was used with daily washing of the chest wall area; and in the study arm, topical Alpha ointment was used in a similar manner. Detailed instructions on topical hydrocortisone cream (1%) and topical Alpha ointment were given to each patient as follows: the patients in both arms were instructed to apply a thin layer of the topical agents twice a day over the chest wall field, beginning on the day of the last session of radiotherapy and continuing every day for 3 weeks. Alpha ointment is a combination of Lawson (natural Henna) and unsaturated fatty acids. Lawson is the main component of Alpha ointment extracted from Lawsonia inermis. All of the patients were followed up for 3 weeks after developing radiation dermatitis. During this period, each patient was examined every week. Dermatitis grade, patients’ age, breast cancer stage, and dermatitis area (cm^2^) were recorded in our data sheet before the commencement of intervention. Subsequently, the dermatitis area (cm^2^) was measured independently by 2 physicians in each examination until 3 weeks after the start of intervention. Irregular dermatitis area was estimated in each patient using a grid paper. The patients’ complaints, including skin burning, pain, and pruritus, and amount of skin discharge change were scored from 0 to 3 in our data sheet in each examination (subjective scores were defined as: 0: no complaint; 1: mild; 2: moderate; and 3: severe complaint). In this study, the rate of dermatitis healing was the objective criterion. The primary end point of the study was the speed of dermatitis healing. Dermatitis healing was defined as complete re-epithelialization of moist desquamation (dermatitis grades 2 and 3) areas. The healing rate of dermatitis (grades 2 and 3) was measured by comparing the rate of the decrease in the dermatitis area (cm/week) between the study and control arms. The mean dermatitis area (cm/week) was compared between the study and control arms during 3 consecutive weeks of intervention. A minimum sample size required 24 patients in each arm to ensure 80% power at the 5% significance level for detecting a 40% improvement in the healing rate from 30% to 70%.

The data were analyzed using statistical tests. The Chi-square test was employed to compare the data percentages at the beginning of the treatment such as age, radiotherapy dose, and stage of disease. The Mann Whitney test was used to compare the changes in the patients’ complaints such as pain and pruritus. And, the *t* test with Bonferroni correction was used to compare burn area dermatitis by SPSS (version 17.0) and to compare the clinical measurements and the clinicopathological characteristics between the trial arms. A P value less than 0.05 was considered statistically significant.

## Results

**Figure 2:**
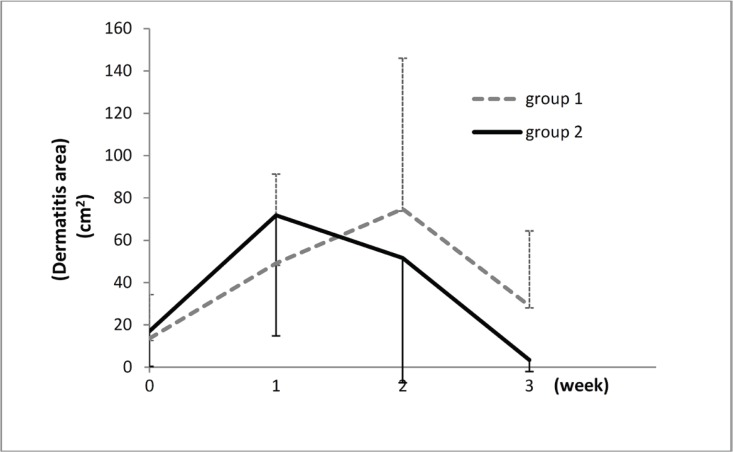
The rate of dermatitis healing time during 3 consecutive weeks of treatment is illustrated here

There was no meaningful difference in terms of baseline variables, including age, sex, dermatitis grade, total radiation dose, disease stage, and dermatitis area (cm^2^) between the two arms. The mean age of the control and study arms was 47 (range=25-72) years and 49 (range=28-81) years, respectively. The mean radiation dose was 49.1 Gy (range=45-50.4 Gy) in the control arm and 48.8 Gy (range=45-50.4 Gy) in the study arm. The mean dermatitis area (summation of grades 2 and 3) was 13.54 (range=0.5-75.0) cm^2 ^in the control arm and 17.02 (range=0.7-78.0) cm^2 ^in the study arm (table 1). All the patients in both arms tolerated the topical treatments well, and no systemic or local reaction or dermatitis aggravation was observed. The analysis of data showed that 3 weeks’ use of topical Alpha ointment twice a day was more effective on the healing of radiation-induced dermatitis than that of topical hydrocortisone cream (1%) (P=0.001). This effect was significant in the second week (P=0.007); however, this difference was not significant for grade 2 dermatitis (P=0.343). This effect was also significant on the healing of grade 3 dermatitis (P=0.003) during 3 weeks of intervention (figure 2, table 2). Furthermore, the healing of both grade 2 and grade 3 dermatitis was significant in the second week of treatment (P=0.027 and P=0.004, respectively). Regarding the patients’ subjective complaints, although there was no statistically significant difference in burning sensation between the two arms over the 3-week intervention period (P=0.078), Alpha ointment decreased the patients’ other complaints such as pain (P<0.001), pruritus (P=0.009), and discharge (P=0.010) effectively and meaningfully. This difference was significant for pain relief in the second and third weeks of treatment (P=0.010 and P=0.016, respectively) and non-significant in the first week (table 3).

**Table 1:** Distribution of baseline variables between the study and control arms at the beginning of intervention

**Variables**	**Control arm**	**Study arm**	**P value**
Mean age (age range)	47.03 (25-72)	49.03 (28-81)	0.507
Mean dermatitis area (cm^2^)			
Grade 2 (±SD)	12.29 (±19.04)	13.02 (±15.96)	0.847
Grade 3 (±SD)	1.25 (±4.76)	4.00 (±8.44)	0.283
Dermatitis grade			
Grade 2	25 (83.3%)	22 (73.3%)	0.530
Grade 3	5 (16.7%)	8 (26.7%)	0.531
Mean RT dose (Gy) (range)	49.1(45-50.4)	48.8 (45-50.4)	0.684

RT: Radiotherapy

**Table 2:** Rate of dermatitis healing during 3 consecutive weeks of treatment

**Radiation dermatitis **	**Control arm (cm** ^2^ **)**	**Study arm (cm** ^2^ **)**		**P value**
Mean area of grade 2 (±SD)				
Baseline	12.29±19.04	13.02±15.96	0.847	
First week	33.17±35.73	29.72±41.22	0.361	
Second week	41.70±53.86	24.07±34.25	0.027	
Third week	17.04±30.23	2.70±4.16	0.108	
Mean area of grade 3 (±SD)				
Baseline	1.25±4.76	4.00±8.44	0.283	
First week	15.93±29.18	42.02±50.15	0.130	
Second week	33.07±57.49	27.58±48.27	0.004	
Third week	11.95±23.29	0.70±2.59	0.719	
Mean area of grade 2+3 (±SD)				
Baseline	13.54±20.76	17.02±16.66	0.436	
First week	49.11±42.14	71.74±56.96	0.137	
Second week	74.77±71.20	51.64±59.04	0.007	
Third week	29.00±35.35	3.39±5.51	0.376	

**Table 3:** Patients’ relief of complaints during 3 consecutive weeks of treatment

**Variables**	**Mean complaint score**		**P value **
	**Study arm**	**Control arm**	
Skin burn relief (±SD)			
Baseline	3.13±0.681	2.93±0.592	0.063
First week	3.30±0.651	3.10±0.548	0.060
Second week	2.50±0.820	2.90±0.759	0.074
Third week	1.53±0.571	1.99±0.694	0.078
Skin pain relief (±SD)			
Baseline	2.77±0.679	2.47±0.507	0.088
First week	3.10±0.607	2.93±0.691	0.324
Second week	2.17±0.747	2.60±0.724	0.026
Third week	1.27±0.450	2.20±0.761	<0.001
Skin pruritus relief (±SD)			
Baseline	2.37±0.718	2.13±0.680	0.741
First week	2.53±0.629	2.43±0.774	0.227
Second week	2.00±0.695	2.23±0.728	0.438
Third week	1.43±0.568	1.87±0.730	0.013
Skin discharge relief (±SD)			
Baseline	1.27±0.450	1.10±0.403	0.136
First week	1.83±0.531	1.67±0.802	0.545
Second week	1.67±0.479	1.77±0.774	0.166
Third week	1.10±0.305	1.37±0.556	0.025

## Discussion

Locally advanced breast cancer accounts for a considerable portion of all breast cancers, particularly in developing countries such as Iran. Nearly all patients with locally advanced breast cancer need to receive radiotherapy as an essential part of their local treatment.^1^^,^^12^ Post-mastectomy radiotherapy can inevitably lead to degrees of dermatitis, affecting the patients’ life quality. Radiation-induced dermatitis may be acute or chronic. Acute radiation dermatitis presents as skin pain, erythema, edema, dry or wet scaling, discharge, pruritus, ulceration, bleeding, and necrosis.^1^^,^^2^ These signs and symptoms usually peak at one to 3 weeks following the last radiation session and relieve within 3-6 weeks of the completion of radiotherapy in dry desquamation. Nevertheless, in cases with moist desquamation (grades 2 and 3), it is prolonged to 4-6 weeks. This acute dermatitis can cause temporary or even complete radiation interruption.^1^^-^^4^^,^^13^ In the present study, acute dermatitis was maximized in the second week in the study arm and in the third week in the control arm. 

In this study, the grades of dermatitis were determined according to the Common Terminology Criteria for Adverse Events (version 4.0).^11^ These scoring criteria are based on visual inspection. This objective measurement assesses the skin toxicity regardless of the patient factors influencing the degree of radiation dermatitis. Yamazaki et al.^14^ evaluated the effect of patient factors on radiation dermatitis in patients with breast cancer who underwent postoperative radiotherapy after breast-conserving surgery. They compared skin color changes (using an objective analyzer) between treated and contralateral normal breasts and found more reddish and higher degrees of radiation dermatitis in heavier patients. Different assessment methods have been drawn upon in other studies.^15^ Yoshikawa et al.^16^ assessed the degree of radiation dermatitis by comparing serial skin change in photographs. In the current study, the primary end point was the rate of wound healing between the study and the control arm; therefore, the assessment method was not an important issue. 

There are various treatment-related and patient-related factors affecting the degree of radiation dermatitis. Any type of recent or old damage due to surgery, burn, or lesions can potentiate radiation dermatitis. Other patient-related factors include history of collagen vascular diseases, diabetes mellitus, renal failure, older age, and concurrent use of immunosuppressive or chemotherapeutic agents. The site of radiotherapy, radiation field size, total dose of radiation, dose per fraction, and type and energy of radiation are considered as treatment-related factors affecting the degree of radiation dermatitis.^17^

There is no consensus regarding the optimal treatment or prevention for radiation dermatitis. Be that as it may, some supportive care, including gentle washing with mild soap, wearing loose cotton clothing, avoiding extreme temperatures, avoiding sun exposure to radiation fields, avoiding shaving or hair removal in radiation fields, and avoiding use of any unproven topical agents like cosmetic products, is generally advised for all patients undergoing radiotherapy.^1^^,^^2^^,^^4^^,^^14^

In the literature, a wide variety of topical agents such as corticosteroids, Aloe Vera, Biafine cream, hyaluronic acid, Sucralfate, Dexpanthenol, and vitamin E have been used in acute radiation-induced dermatitis. Nonetheless, the existing evidence is insufficient to recommend the use of a specific topical agent to prevent or to treat this complication.^1^^-^^4^^,^^17^^,^^18^ Therefore, systematic reviews suggest that the efficacy of the agents and approaches should be compared in phase I and II clinical trials.^2^^,^^17^

One of the proposed treatments for radiation-induced dermatitis is the use of topical corticosteroids. Anti-inflammatory effects of these agents may play an important role in relieving patients’ symptoms.^1^^-^^4^^,^^17^^,^^18^ Some evidence indicates the moisturizing effects of hydrocortisone cream as the likely mechanism in the healing of radiation-induced dermatitis. Moisturization plays an essential role in the early prevention of acute dermatitis. According to this mechanism, hydrophilic agents such as Aloe Vera gel or vegetable oil reduce the severity of radiation dermatitis as well as topical hydrocortisone.^13^^,^^19^ There is no clear evidence to support the superiority of potent corticosteroids over hydrocortisone in the literature. In a study, Clobetasone butyrate caused more severe radiation reactions compared to hydrocortisone with similar prescribed radiation doses.^20^ In another study, Schmuth et al.^18^ compared the topical cream of hydrocortisone (1%) and the topical cream of Dexpanthenol (0.5%) in the healing of acute radiation-induced dermatitis; however, they found no significant difference in dermatitis healing between the two treatment arms. Other local treatments such as Dexpanthenol, Calendula, and honey ointment have been used for the treatment of dermatitis in different studies.^1^^,^^2^^,^^4^^,^^17^^,^^21^ Another drug which has newly been introduced for the management of burn and infectious wounds is natural Henna (Lawsonia inermis linn), as was used in our study in the form of “Alpha ointment”.^7^^-^^10^ Many of today’s modern drugs have their origin in traditional plant medicine. Alpha ointment is a combination of Lawson (natural Henna) and unsaturated fatty acids. Lawson is the main component of Alpha ointment extracted from Lawsonia inermis. Unsaturated fatty acids in Alpha ointment have an anti-inflammatory role.^13^ There is some evidence supporting the safety and efficacy of natural henna in wound healing.^8^^,^^10^^,^^22^^-^^24^ In a study conducted by Gunjan Guha et al.^7^ antioxidant effects of Henna derivatives were proved. In an animal study, Nayak et al.^8^ demonstrated the wound healing activity of natural henna using excision, incision, and dead space wound models. Philip Jacob et al.^9^ in recent study found antioxidant and scavenging activity of Henna derivatives. In addition, Hoseini et al.^10^ showed further effectiveness of Alpha ointment (Henna-bearing ointment) in comparison with topical silver Sulfadiazine in grade 3 burn wounds infected by *Pseudomonas aeruginosa*. Yucel and Guzin^24^ observed the efficacy and safety of natural Henna in treating hand-foot syndrome in 10 patients with colon and breast cancer following chemotherapy with Capecitabine alone or combined with Docetaxel. In our study, topical Alpha ointment (containing natural henna) was more effective on the healing of radiation-induced dermatitis than was topical hydrocortisone cream (1%) in the second week of intervention. In addition, Alpha ointment significantly decreased the patients’ complaints such as pain, pruritus, and discharge compared to topical hydrocortisone cream (1%). 

Henna is an inexpensive natural plant agent with anti-inflammatory, antipyretic, and analgesic effects. It also plays an antioxidant and immunomodulatory role and lacks the potential acute and late adverse effects of corticosteroids.^24^^,^^25^

To date, there has been no recorded study investigating the efficacy of Alpha ointment in the healing of acute radiation-induced dermatitis. The current study had some limitations in terms of time and financial issues. The sample size was small and the length of patients’ follow-up was limited. 

## Conclusion

According to the results of this study, three weeks’ use of topical Alpha ointment was more effective on the healing of radiation-induced dermatitis than was topical hydrocortisone cream (1%) in our breast cancer patients. Further evaluation with larger numbers of patients is suggested for the confirmation of these preliminary results.
